# Strangulated internal hernia via a congenital posterior gastric antral mesenteric defect in an adolescent: a rare case report and literature review

**DOI:** 10.1186/s12893-025-03334-5

**Published:** 2025-12-30

**Authors:** Kangnan He, Yunjie Ju, Ping Cheng, Peng Xu

**Affiliations:** 1 Department of Urology, Union Hospital, Tongji Medical College, Huazhong University of Science and Technology, Wuhan, 430022 China; 2 Department of Emergency, Union Hospital, Tongji Medical College, Huazhong University of Science and Technology, Wuhan, 430022 China

**Keywords:** Congenital mesenteric defect posterior to the gastric antrum, Transmesenteric hernia, Internal hernia, Side-to-side anastomosis, Acute abdomen, Adolescent

## Abstract

**Background:**

Congenital transmesenteric hernia, an uncommon cause of acute abdomen with an incidence of less than 1%, is often identified only intraoperatively.We present a rare case of internal hernia through a congenital mesenteric defect posterior to the gastric antrum, precipitated by strenuous exercise after a meal in an adolescent.

**Case presentation:**

A 17-year-old male developed sudden severe abdominal pain and distension after vigorous exercise following a meal. Abdominal computed tomography (CT) demonstrated an abnormally displaced duodenal loop, raising suspicion for internal herniation. Laparoscopic exploration revealed that a large segment of the small bowel had herniated through a congenital mesenteric defect posterior to the gastric antrum into the right upper abdomen. The herniated bowel exhibited clockwise torsion, forming a closed-loop obstruction with strangulated necrosis. Due to extensive necrosis and limited working space, the procedure was converted to an open laparotomy for definitive resection. Following resection of the necrotic bowel, only 6 cm of terminal ileum remained. To preserve the ileocecal valve function, after the viability of both ends was confirmed and with informed consent from the patient’s guardians, an ileo-ileal side-to-side anastomosis was performed to restore intestinal continuity. By postoperative day 7, pelvic drainage was clear, without evidence of anastomotic leakage, and the patient had a smooth recovery and was subsequently discharged.

**Conclusion:**

Emergency physicians should maintain a high index of suspicion for transmesenteric hernia as a potential cause of acute strangulation and bowel necrosis in patients presenting with acute abdomen, and timely surgical intervention is essential. Although long-term outcomes require further evaluation, ileal resection near the ileocecal region with preservation of the valve using a side-to-side anastomosis may represent a salvage strategy under high-risk anatomical conditions.

## Introduction

Internal hernia (IH) is defined as the protrusion of intra-abdominal viscera through congenital or acquired defects of the peritoneum or mesentery into abnormal anatomical spaces [1, 2]. The anatomical basis may involve congenital structural anomalies, such as the foramen of Winslow, paraduodenal recesses, or intersigmoid fossa, as well as secondary defects caused by trauma, inflammation, or previous surgery [2]. As a rare cause of intestinal obstruction, IH has an overall incidence of less than 1% [3]. Its clinical manifestations are often nonspecific, and diagnosis is frequently established only during surgery [4, 5]. Because IH may lead to bowel obstruction and strangulation with subsequent necrosis, delayed diagnosis can result in a mortality rate of up to 70% [6, 7]. Here, we report the case of a 17-year-old male who presented with acute abdominal pain and distension and was ultimately diagnosed with suspected congenital transmesenteric hernia complicated by strangulated bowel obstruction. Through analysis of this case, we aim to provide insights for emergency surgical decision-making and to explore the potential mechanisms of bowel necrosis and surgical intervention strategies.

### Clinical data

A 17-year-old male was admitted to the emergency department with a 24-hour history of persistent abdominal pain and distension. The symptoms began after vigorous exercise following a meal, presenting as sudden colicky epigastric pain with progressive abdominal distension. Abdominal CT at a local hospital revealed abnormal duodenal positioning, dilated and gas-filled bowel loops in the left upper abdomen, bowel wall thickening, and the transmesenteric “whirl sign,” suggesting paraduodenal hernia (PDH) with small-bowel volvulus. Conservative treatment with nasogastric decompression and intravenous fluid resuscitation was ineffective, and the patient was transferred to our hospital.

**Fig. 1:**
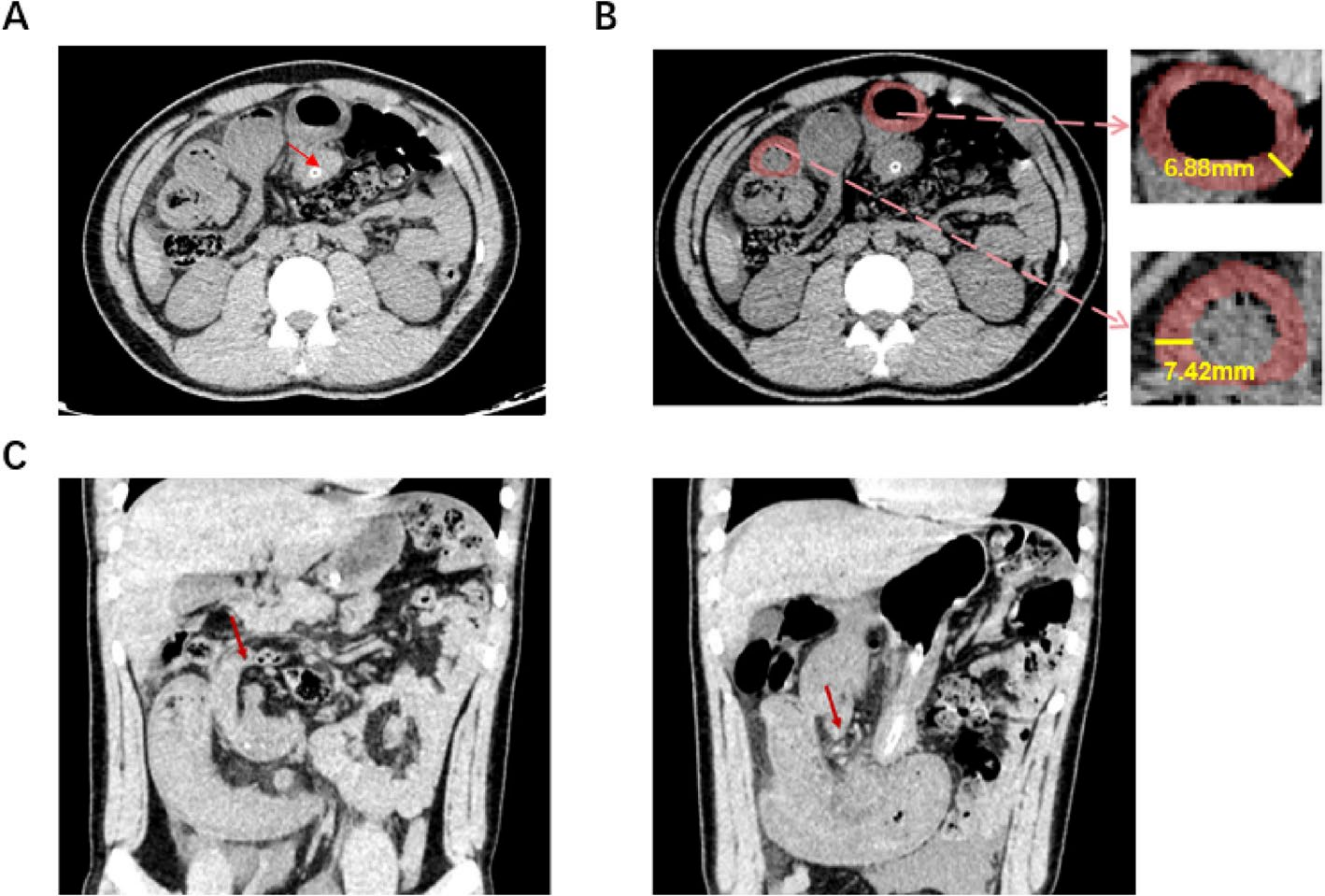
Preoperative abdominal CT findings of an internal hernia through a congenital mesenteric defect posterior to the gastric antrum with strangulated small bowel. **A** Axial CT showing an abnormally positioned small bowel loop with focal dilatation (red arrow). **B** Axial CT demonstrating the herniated small bowel loop with thickened bowel wall and luminal narrowing. Magnified views reveal bowel wall edema, with measured thicknesses of 6.88 mm and 7.42 mm. **C** Coronal CT further showing the terminal ileum herniated into the right upper abdominal cavity (red arrow), accompanied by obstruction and edema

On admission, he appeared distressed, with abdominal distension, significant diffuse tenderness, and mild rebound tenderness on examination. Hemodynamic assessment revealed a heart rate of 85 beats per minute and a blood pressure of 125/85 mmHg. Laboratory tests showed a white blood cell count of 10.38 × 10⁹/L with neutrophilia (9 × 10⁹/L).The patient's red blood cell count was slightly low at 4.09 × 10^12^/L, and hemoglobin was at the lower limit of normal at 125 g/L. Inflammatory markers were markedly elevated, with a C-reactive protein of 215 mg/L and procalcitonin of 11.71 ng/mL, suggesting a significant systemic inflammatory response to infection. Repeat CT scan confirmed intestinal volvulus. Based on these findings, the patient was admitted with a provisional diagnosis of intestinal obstruction and acute diffuse peritonitis. (Fig. 1) He reported no changes in bowel habits and had no remarkable past medical, family, or medication history.

### Surgical procedure and outcome

Emergency laparoscopy revealed a large segment of small bowel herniating in an antecolic fashion through a congenital mesenteric defect posterior to the gastric antrum into the right upper abdomen.The herniated bowel was twisted clockwise, forming a closed-loop obstruction with necrosis, and about 1000 mL of hemorrhagic ascites was present in the peritoneal cavity. The procedure was subsequently converted to a laparotomy via a right rectus abdominis incision. After adhesiolysis and reduction of the herniated bowel, a 40-cm segment of the terminal ileum was identified as non-viable. This necrotic segment was subsequently resected. Following resection, only 6 cm of viable terminal ileum proximal to the ileocecal valve remained.Intraoperative assessment demonstrated black-purple discoloration, absent peristalsis, and no transmesenteric pulsation, confirming the bowel was non-viable. A 40-cm necrotic segment was resected using a linear stapler (disposable powered endoscopic linear cutting stapler and components, Model HLEFL45; Jiangxi Hanliang Biotechnology Co., Ltd.), with transections performed 5 cm beyond the necrotic margins.

**Fig. 2:**
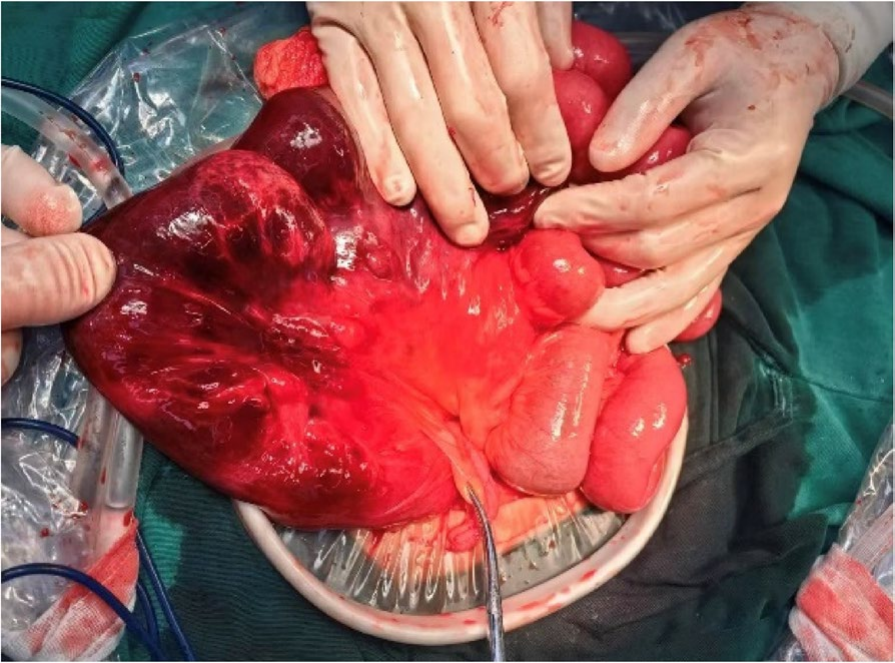
Intraoperative findings of a patient with an internal hernia through a congenital mesenteric defect posterior to the gastric antrum, complicated by small bowel necrosis. Laparotomy revealing approximately 40 cm of necrotic bowel, with the clamped site identified as the appendix

Given that only 6 cm of terminal ileum remained, preservation of the ileocecal valve was prioritized. After detailed intraoperative discussion with the patient’s family, a modified side-to-side anastomosis was performed: (i) seromuscular traction sutures were applied to approximate the proximal and distal bowel segments; (ii) longitudinal enterotomies were made 1 cm from each transected end, and a functional side-to-side anastomosis was constructed with a linear stapler; (iii) the anastomosis was reinforced with continuous 3-0 absorbable sutures, ensuring patency for one finger; (iv) meticulous hemostasis was achieved, the transmesenteric defect was closed, and a pelvic drain was placed. Intraoperative image is shown in Fig. 2.

Histopathological examination confirmed mucosal atrophy and transmural necrosis with vascular congestion, consistent with hemorrhagic infarction secondary to volvulus. Postoperatively, the patient recovered uneventfully. By day 7, the drainage was clear, inflammatory markers had significantly improved, and no signs of anastomotic leakage were observed. He was discharged in good condition. At 3-month follow-up, he reported full recovery, with normal diet, regular bowel habits, and no specific complaints.

## Discussion

Previous studies have classified congenital internal hernias into intraperitoneal and retroperitoneal subtypes, with rarer variants such as paravesical or transanal hernias also reported [8]. Congenital transmesenteric defects often occur in avascular regions of the mesentery. In 1836, Rokitansky first described transmesenteric hernia in a cadaveric study, with an incidence of 0.5%–6% among autopsies of patients with intestinal obstruction [9]. In more recent years, transmesenteric hernias are more commonly encountered as complications of laparoscopic Roux-en-Y gastric bypass or gastrectomy, whereas congenital cases remain extremely rare, usually occurring in children. Their clinical presentation is nonspecific, including nausea, vomiting, abdominal pain, and distension, and diagnosis is often delayed until the development of obstruction, strangulation, or necrosis, with mortality rates remaining high.

**Fig. 3:**
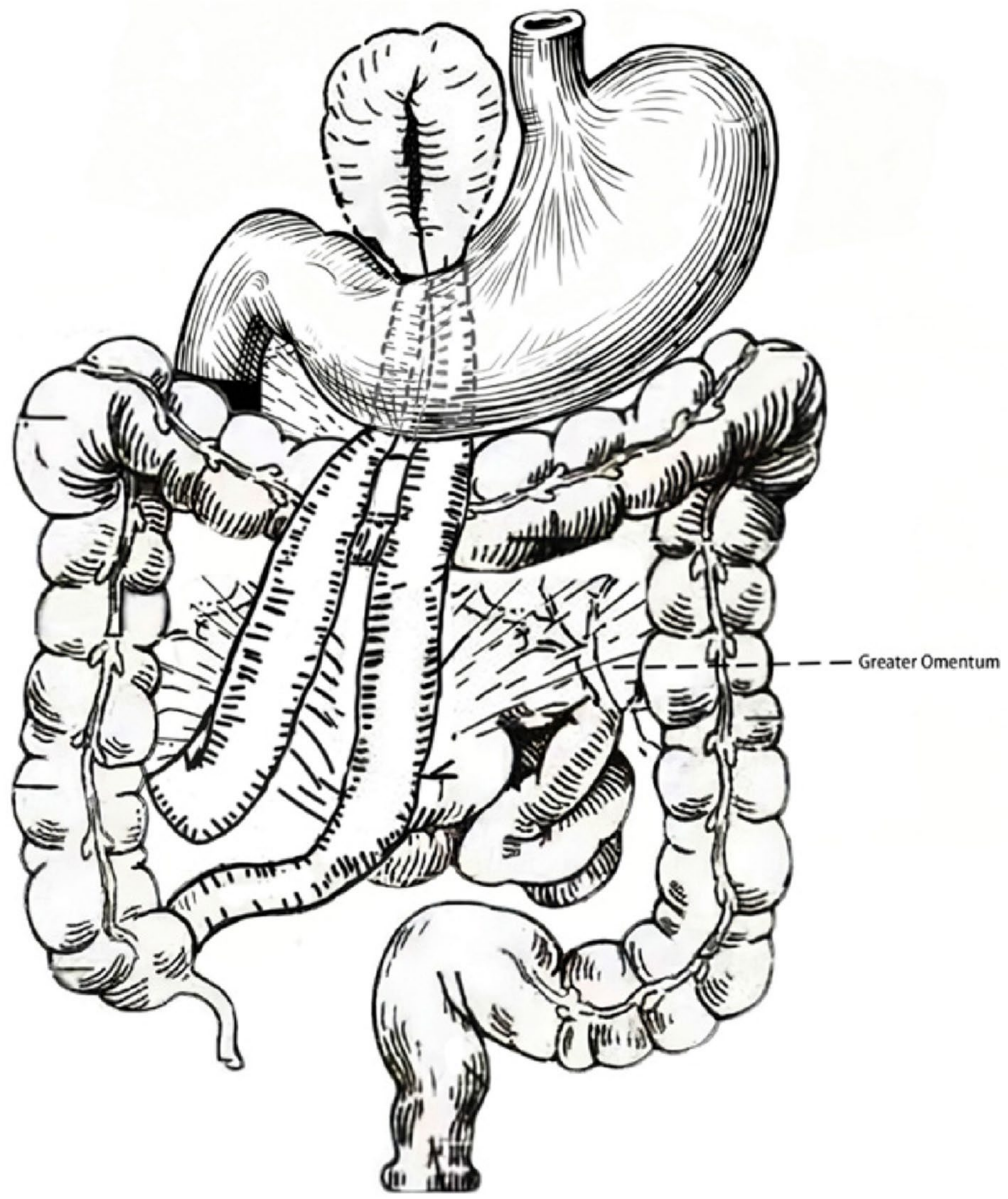
Hand-drawn anatomical illustration showing the gastric antrum, colon, and greater omentum. The congenital mesenteric defect in this case was located posterior to the gastric antrum, an atypical site that contributed to the difficulty of preoperative diagnosis

In the present case, preoperative CT suggested a paraduodenal hernia, the most common type of congenital internal hernia [10]. Typical left paraduodenal hernias arise from Landzert’s fossa, with the hernia sac located to the left of the fourth part of the duodenum and posterior to the mesocolon, bounded by the inferior mesenteric vein and the left branch of the middle colic artery. Right-sided types originate from Waldeyer’s fossa, located at a defect in the first jejunal mesentery [11]. However, the hernia orifice in this case was located posterior to the gastric antrum, which deviated significantly from typical PDH or Winslow’s foramen hernias. The foramen of Winslow is the passage between the greater and lesser peritoneal sacs, which is anatomically bounded anteriorly by the free right margin of the hepatoduodenal ligament, posteriorly by the peritoneum overlying the inferior vena cava, superiorly by the caudate lobe of the liver, and inferiorly by structures including the first part of the duodenum and the hepatic artery [12]. Its typical pathway and imaging findings are relatively well-defined. In the present case, because the hernial orifice was located posterior to the stomach, this atypical anatomical position significantly increased the difficulty of preoperative radiologic diagnosis. Although CT is the preferred modality for early detection of internal hernia, its diagnostic value relies heavily on recognition of typical locations. In non-classical cases, imaging may reveal only indirect signs such as bowel dilatation, mural thickening, or the “small-bowel feces sign” [13]. In our case, CT suggested PDH, yet intraoperative exploration confirmed a unique internal hernia through a congenital mesenteric defect posterior to the gastric antrum, highlighting the importance of surgical exploration for definitive diagnosis. It is noteworthy that while the initial CT report from the local hospital suggested abnormal duodenal positioning, this was not confirmed during our surgical exploration, where the duodenum was found to be in its normal anatomical location. We hypothesize that the mass effect created by the large segment of herniated small bowel aggregating in the right upper abdomen, coupled with localized inflammation from the gangrenous tissue, likely compressed adjacent structures, thus mimicking the appearance of a displaced duodenum on the CT scan. This discrepancy further underscores the limitations of preoperative imaging in complex and atypical hernia cases.We provide a schematic anatomical illustration highlighting the unusual location of the congenital mesenteric defect posterior to the gastric antrum (Fig. 3).

The pathogenesis of congenital transmesenteric defects remains unclear, but may involve ischemic changes of the embryonic mesentery, abnormal regression of the dorsal mesentery, or mechanical injury during intestinal development [8]. While 28 cases of congenital IH have been reported in pediatric patients [8], adult cases remain extremely rare. The uniqueness of our case lies in its onset immediately after strenuous postprandial exercise. We hypothesize that the mechanism involves gastric distension after food intake, which may have exposed a latent weak point posterior to the gastric antrum. The subsequent abrupt rise in intra-abdominal pressure during vigorous exercise likely forced excessively mobile bowel loops into this potential space, resulting in torsion, closed-loop obstruction, and rapid progression to necrosis, as observed intraoperatively. This pathophysiological process is comparable to the case described by Brezler et al. [14], in which a child with congenital malrotation and a mesocolic defect developed midgut volvulus and catastrophic internal herniation. In that case, nearly the entire small intestine and part of the colon herniated through a transmesenteric defect and underwent a 900-degree clockwise volvulus. Despite emergency surgery, delayed presentation and severe ischemia–reperfusion injury necessitated multiple reoperations, during which a total of 410 cm of necrotic bowel was resected. Consequently, the patient developed short bowel syndrome and became dependent on total parenteral nutrition. This cascade of events further illustrates how, in individuals with congenital anatomical abnormalities, physiological or behavioral factors may act as critical triggers for the onset of acute abdomen.

A major intraoperative challenge in our case was the extremely short residual ileum (6 cm) following resection of approximately 40 cm of necrotic bowel. Several options were considered: (i) ileocolic anastomosis, which provides a secure anastomosis on well-perfused tissue but sacrifices ileocecal valve function and increases the risk of diarrhea and malabsorption; (ii) an end ileostomy, which would have been a safe choice in the presence of severe intra-abdominal infection and hemodynamic instability, consistent with the principles of damage control surgery; and (iii) an ileocecal valve-preserving anastomosis, which was the salvage strategy ultimately adopted in this case.Although carrying certain risks, this option maximizes preservation of intestinal continuity and physiological barrier function. A side-to-side anastomosis was chosen over an end-to-end anastomosis because it provides a wider lumen, reduces the risk of stricture, and can be performed in a shorter operative time [15]. A retrospective study including 69 patients undergoing Natural Orifice Specimen Extraction Surgery (NOSES) for left-sided colon cancer demonstrated that end-to-end anastomosis may lead to mild stricture due to scar formation. In contrast, side-to-side anastomosis, often preferred in techniques like NOSES for its wider anastomotic area and smoother mucosal healing, theoretically reduces the risk of narrowing [15]. Moreover, the operative time for side-to-side anastomosis was significantly shorter than that of end-to-end anastomosis (17.2 ± 1.6 min vs. 27.8 ± 2.7 min, P < 0.001). In the present case, this strategy proved successful: no anastomotic leak occurred, and at 3-month follow-up, the patient exhibited no signs of nutritional deficiency or malabsorption. These findings suggest that, under high-risk anatomical conditions, this approach is feasible. Future studies with larger case series will be necessary to further evaluate its long-term prognosis, functional recovery, and potential complications.

In summary, this case reports a rare internal hernia occurring in the congenital mesenteric defect posterior to the gastric antrum, triggered by strenuous exercise after a meal. Its atypical anatomical location increased the difficulty of preoperative imaging diagnosis and underscores the importance of maintaining a high index of suspicion in young patients presenting with acute abdomen without prior surgical history. Review of pediatric cases in the literature, such as the occult neonatal bilious vomiting described by Batsis et al. [16] nd the catastrophic short bowel syndrome reported by Brezler et al. [14], consistently highlights a central theme: early surgical intervention is the decisive factor for prognosis.

Based on previous reports and our institutional experience, we propose the following strategies: (i) clinicians should maintain vigilance for internal hernia in young patients without a history of abdominal surgery, particularly in those with recurrent or unexplained abdominal pain, and include it in the differential diagnosis; (ii) when clinical symptoms are suggestive but imaging findings are inconclusive,s early diagnostic laparoscopy should be strongly considered, as overreliance on imaging may delay timely surgical exploration. Such an approach not only enables salvage of ischemic bowel but also represents an effective measure to reduce the morbidity and mortality associated with this rare entity.

## Data Availability

The datasets and materials supporting this case report are available from the corresponding author upon reasonable request.
